# Phylogenetic and modelling analysis of purple acid phosphatase 18 (SiPAP18) from Setaria italica

**DOI:** 10.6026/97320630017727

**Published:** 2021-08-31

**Authors:** Chinreddy Subramanyam Reddy, Tanushri Kaul, Khaled Fathy Abdel Motelb, Sonia Khan Sony, Jyotsna Bharti, Rachana Verma

**Affiliations:** 1 Department of Botany, CSSR and SRRM Degree College, Kamalapuram, Kadapa District, Andhra Pradesh-516219, India; 2 Nutritional Improvement of Crops Group, International Center for Genetic Engineering and Biotechnology, New Delhi, India

**Keywords:** Purple acid phosphatase, protein modelling, phylogenetic analysis, phosphorus accessibility

## Abstract

Purple acid phosphatases belong to metallo-phosphatase family. Intracellular phosphatases are crucial for phosphorus (P) distribution in the cell and are highly induced in phosphorus-deprived conditions in the soil. Disparate PAP isoforms exist within discrete subcellular compartments in Setaria italica and their expression in P deprived conditions fosters phosphorus amelioration. We isolated the SiPAP18 gene and developed the homology SiPAP18 protein model based on the crystal structure of the Kidney bean PvPAP (PDB ID: 2QFP) as template (sequence similarity 42.7%) using Modeller 9.12 with adequate validation. Structure model analysis shows the significance of five conserved signatures with seven metal-paired amino acid residues during P-deprivation induced phosphorus amelioration.

## Background:

Phosphorus (P) is a vital macronutrient, basic component of nucleic acid and phopholipids. It is crucial for numerous enzymatic and biochemical pathways in plants, such as photosynthesis, nucleotide synthesis, membrane remodelling, and protein modification [1]. Large amount of P is available in the soil in the form of organic and inorganic P complexes. However, it is not accessible for plants, particularly in acidic soils [2-3]. Nevertheless, Pi supplementation is crucial for plant growth and development. Simultaneously, sustainable utilization of fertilizer P aids in forestalling the undesirable P-effluence and its ecological effects on the wider environment [4-6]. Hence, breeding cultivars with high P-efficiency and optimized field P management practices are usual for sustainable agricultural development [7].

Purple Acid Phosphatases (PAPs) are prerequisite for sustainable Pi mobilization and P utilization from soil is needed for the sustenance of plants [6]. PAPs are active at acidic pH and can hydrolyze broad range of phosphoric acids and anhydrides [8]. It should be noted that the term Purple Acid Phosphatase is derived from a charge transfer transition between tyrosine to chromo ferric Fe (III) in the di-nuclear metal center. The signature motifs of these proteins are comprised of five blocks with seven residues essential for metal synchronization [9-10]. PAPs have been categorized as low molecular monomer proteins with (LMM) approximately 35 kD and high molecular oligomeric PAPs, with a subunit mass of approximately 55 kD [5]. Therefore, it is of interest to document the phylogenetic and modelling analyses of purple acid phosphatase 18 (SiPAP18) from Setaria italica.

## Materials & Methods:

### Sequence identification:

SiPAP18 gene was downloaded from National Center for Biotechnology Information (NCBI). PgPAP19 with phytase and PAP activity resembled the sequence of PAP18 (XP_004982542.1) from Setaria italica. Protein modelling was completed using standard homology modelling [11]. Orthologous sequences for SiPAP18 were searched using the Basic Local Alignment Search Tool (BLAST) [12] against the Protein Database (PDB). The X-ray structure with PDB ID: 2QFP for PAP from kidney bean with 42.7 % similarity to our target protein SiPAP18 was selected from the BLAST results.

### Model prediction:

The structure model of SiPAP18 from Setaria italica was created using the Modeller 9.12 software. The MODELLER software implements relative protein structure prediction using structural restraints with known data.

### Model validation of SiPAP18:

The predicted protein model was validated for geometrical and stereo-chemical constraints using tools such as PROCHECK and ProSA-Web [13].

### Phylogenetic analysis:

Phylogenetic analysis of the sequences was developed using the UPGMA method available in the Molecular Evolutionary Genetic Analysis (MEGA) software (version 4.0.02) [9]. Each node was tested using the bootstrap approach by taking 1,000 replicates.

## Results and Discussion:

**Figure 1:**
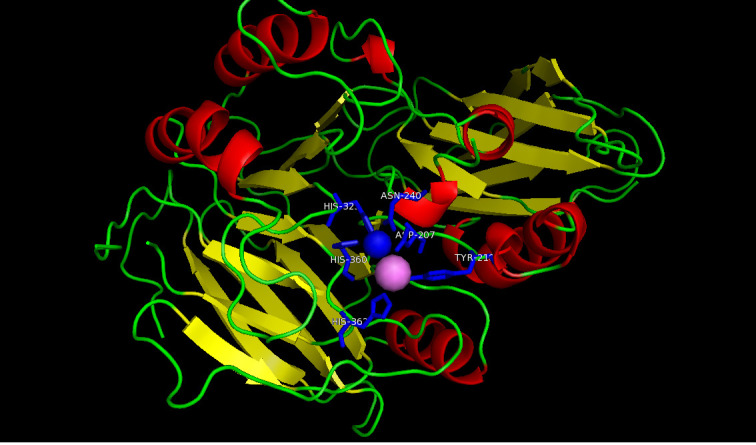
3D model of Si PAP18 with ligand binding sites developed using the PDB template for kidney bean with PDB ID: 2QFP.

The foxtail millet protein sequence comprised of 472 amino acid residues. The homology search using PDB blast picked a target with 42 % sequence identity to SiPAP18 from kidney bean (PDB ID: 2QFP) with an e-value of 6e-153. The ScanProsite server identified the metal ligating string of seven amino acid residues RHGXRXP as the consensus pattern of signature motif for purple acid phosphates in both foxtail millet and kidney bean PAPs. Sequence alignment showed another signature motif that comprised of five metal binding domains conserved in both target and template PAP sequences (Figure 1). We also developed the structural models for SiPAP18 using Modeller 9.12. The model with the lowest DOPE (Discrete Optimized Protein Energy, a statistical potential used to assess homology models) score of -25107.04 that is considered to be thermodynamically stable was chosen for further refinement and validation. Accelrys Discovery Studio Version 2.5 was used for visualization.

**Figure 2:**
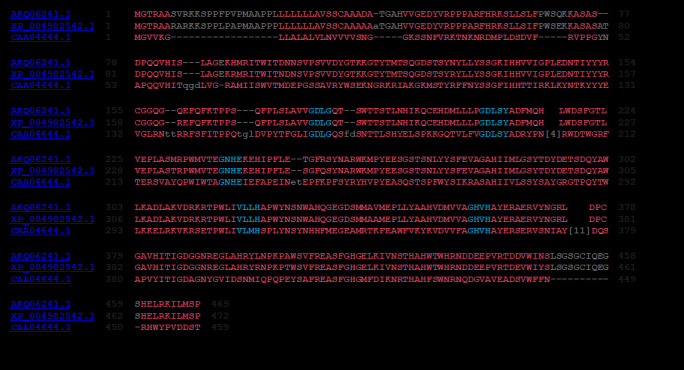
Multiple sequence alignment (MSA) of Pennisetum glaucum PAP18, Setaria italica and Phaseolus vulgaris PAPs with conserved motif (GDLG-xnGDLSY-xn-GNHE/Dxn-VLLH-xn-GHVH) shown in blue colour. SiPAP18 have 95% sequence similarity to PgPAPA18 and 42% to PvPAP.

Data showed that the PAP18 protein shared similarity to the known Pennisetum glaucum PAP18 (95%) assigned as SiPAP18. The full-length gene encoded 472 amino acid residues including an extracellular signal peptide. Data showed the structural similarities of SiPAP18 with PgPAP18 with 95% homology. Sequence analysis showed binuclear metal (Fe+3 -Zn+2) center indicating that SiPAP18 belonged to the family of metallo-phosphatases family. The plant metallo-purple acid phosphatase super-family has been characterized by the presence of two aspartic acid residues (Asp-180-207), one tyrosine (Tyr-210), one asparagine (Asn-240) and three histidines (H-323- 360-362) with five highly conserved motifs (GDLG-xnGDLSY-xn-GNHE/Dxn-VLLH-xn-GHVH) as in PgPAP18 (Figure 2). PAP activity and heat resistance is known in similar proteins. Hence, the molecular details of SiPAP18 are of interest. Thus, the model provides insights into the molecular function of the metal binding residues and phosphatase activity of SiPAP18 in response to P scarcity.

**Figure 3:**
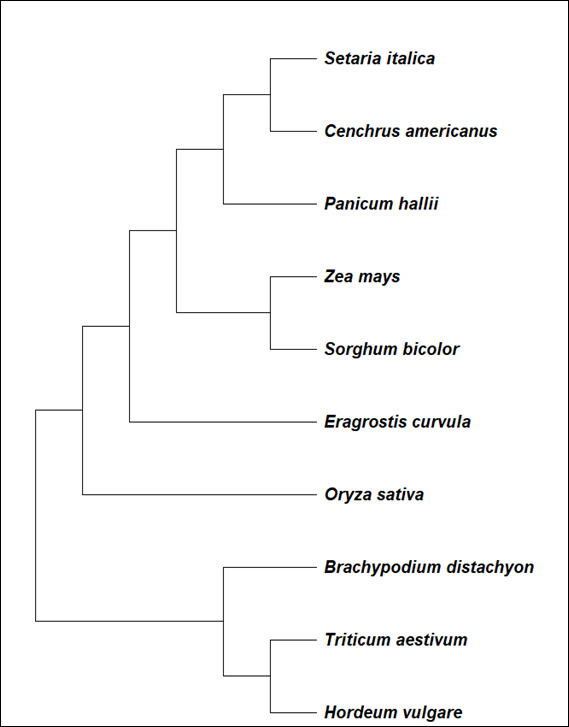
Phylogenetic tree showing the similarity of Setaria italica purple acid phosphatase 18 with other purple acid phosphatase 18 from other plants.

A multigene family that are highly conserved across all cereals encodes the PAP18 proteins. Duplication of genes and their subsequent divergence were central to multiplicity of the PAP gene family. The evolutionary relationship of SiPAP18 with other plant orthologues for PAP18 from closely related 10 species is shown using a phylogenetic tree. The full-length amino acid sequences analysis of SiPAP18 (Acc no. XP_004982542.1) showed 80-95% similarity to other PAP18-like family members (Figure 3). The phylogenetic tree showed that SiPAP18 was 82.9% identical to Hordeum vulgare (Acc. no. BAJ96808.1), Triticum aestivum 83.9% (Acc No: KAF7078064.1), Oryza sativa 85.8% (Acc No: EAY90700.1), Zea mays 88.7% (Acc No: NP001150058.1). Brachypodium distachyon 89.4% (Acc No: KOK14366.1), Sorghum bicolor, 90.7% (Acc no. XP002464327.1), Eragostris curuvla 90.49% (Acc no: TVU46366.1) and Panicum halli 93.8% (Acc no. PUZ38177.1) were used for the construction of the phylogenetic tree. The phylogenetic tree (Figure 3) shows SiPAP18 shared maximum similarity (95%) to Pennisetum glaucum (Acc. no: AKQ06241).

## Conclusion:

We document the phylogenetic and modeling analysis data for the purple acid phosphatase 18 (SiPAP18) from Setaria italica.
